# Significance of eggshell morphology as an additional tool to distinguish species of sand flies (Diptera: Psychodidae: Phlebotominae)

**DOI:** 10.1371/journal.pone.0263268

**Published:** 2022-02-25

**Authors:** Narissara Jariyapan, Pongsri Tippawangkosol, Sriwatapron Sor-Suwan, Chonlada Mano, Thippawan Yasanga, Pradya Somboon, Jérôme Depaquit, Padet Siriyasatien

**Affiliations:** 1 Department of Parasitology, Faculty of Medicine, Chulalongkorn University, Bangkok, Thailand; 2 Vector Biology and Vector Borne Diseases Research Unit, Department of Parasitology, Faculty of Medicine, Chulalongkorn University, Bangkok, Thailand; 3 Department of Parasitology, Faculty of Medicine, Chiang Mai University, Chiang Mai, Thailand; 4 Medical Science Research Equipment Center, Faculty of Medicine, Chiang Mai University, Chiang Mai, Thailand; 5 Faculté de Pharmacie, Université de Reims Champagne-Ardenne, SFR Cap Santé, EA7510 ESCAPE–USC ANSES VECPAR, Reims, France; Universidade Federal da Bahia, BRAZIL

## Abstract

Morphological characteristics of eggshells are important in sand fly ootaxonomy. In this study, eggshells from *Phlebotomus stantoni* Newstead, *Sergentomyia khawi* (Raynal), and *Grassomyia indica* (Theodor) sand flies collected in Chiang Mai province, Thailand were examined and characterized using light microscopy (LM) and scanning electron microscopy (SEM). Then, eggshell morphology of these three species was described for the first time. Each gravid female was forced to lay eggs by decapitation and the eggs were collected for SEM analysis. Egg laying females were identified by morphological examination and molecular typing using *cytochrome* b (*Cyt*b) as a molecular marker. The chorionic sculpturing of *Ph*. *stantoni* eggs combines two patterns on the same egg: unconnected parallel ridges and reticular patterns. *Sergentomyia khawi* and *Gr*. *indica* have similar chorionic polygonal patterns, but their exochorionic morphology and aeropylar area are different. Results indicate that eggshell morphological characteristics such as chorionic pattern, exochorionic morphology, inter-ridge/boundary area, aeropylar area (including the number of aeropyles) and basal layer, can be useful to develop morphological identification keys of eggs. These can serve as an additional tool to distinguish species of sand flies. In addition, the chorionic sculpturing of the eggs of the three species of sand flies observed by LM is useful for species identification in gravid females with spermathecae obscured by eggs.

## Introduction

Phlebotomine sand flies (Diptera: Psychodidae: Phlebotominae) are hematophagous insects. Some species are vectors of several viral, bacterial, and protozoal diseases, especially leishmaniasis. In the last few decades, new proposals for classification and identification of Phlebotominae sand flies have been based primarily on adult morphology [1]. However, morphological identification of the adult stage alone may be insufficient to differentiate closely related species. Hence, an additional approach which uses the characteristics of immature stages, including eggs, has proved useful for identification [2]. In mosquitoes, analyzing morphological characteristics of the chorionic sculpturing of eggs by means of scanning electron microscopy (SEM) can help to differentiate sibling species of the *Anopheles culicifacies* complex [3].

The chorionic sculpturing of sand fly eggs was first studied and described by Ward and Ready in thirteen species of New World sand flies [4]. The ultrastructure of sand fly eggshells was investigated in many New World species (approximately 50 species) [4–19] but only in a limited number of species of Old World sand flies [20–25]. Chorionic patterns reflect phylogenetic relationships among certain *Lutzomyia* sand fly species in the New World [6–8, 10]. The comparison of eggshell morphology of *Nyssomyia intermedia* (Lutz and Neiva) and *Ny*. *whitmani* (Antunes and Coutinho) revealed that chorionic patterns of both species are similar. However, details in the microanatomy of the external surface of the eggs observed by SEM can be used to differentiate the two species [14]. Alencar and Scarpassa [19] examined the chorionic sculpturing of ten Brazilian sand fly species using SEM: *Ny*. *antunesi* (Coutinho), *Ny*. *whitmani* (Antunes and Coutinho), *Bichromomyia flaviscutellata* (Mangabeira), *Bicholmeca nociva* (Young and Arias), *Evandromyia walkeri* (Newstead), *Ev*. *williamsi* (Antunes and Coutinho), *Deanemyia maruaga* (Alves, Freitas and Barrett), *De*. *samueli* (Deane), *Viannamyia furcata* (Mangabeira) and *Lutzomyia dispar* (Martins and Silva). The authors demonstrated that eggshell morphology can be used in phlebotomine taxonomy, both at generic and specific levels.

So far, several chorionic patterns of sand fly eggs have been proposed, *i*.*e*., “polygonal”, “volcano-like” [4], “connected parallel ridges”, “unconnected parallel ridges” [6], “elliptical” [7], “reticular”, “verrucose”, “disperse” [8], and “placoid” [19]. The ultrastructural morphology of eggs of Old World sand flies in the genera *Sergentomyia* and *Phlebotomus* was studied in Italy, Kenya, Ethiopia, Egypt, and India [20–25]. Ward and Ready suggested that chorionic sculpturing differences are adaptations to the different types of environments of oviposition sites [4].

In Thailand, surveillance studies of sand fly species were carried out in different regions of the country over time. At least 31 species of sand flies have been identified [26–29]. Several studies were carried out to detect the DNA/RNA of various pathogens in sand flies [30–35]. DNA of *Leishmania martiniquensis*, *L*. *orientalis*, and *L*. *donovani*/*L*. *infantum* was detected in some species of *Sergentomyia* [30, 32] probably misidentified as *Se*. *gemmea* (Lewis and Jeffery) [28]. Moreover, DNA of *L*. *martiniquensis* was detected in *Se*. *barraudi* (Sinton), *Se*. *khawi*, and *Ph*. *stantoni* [31, 32]. However, the role of sand flies as natural vectors of *L*. *martiniquensis* remain unclear [28]. The DNA of *Trypanosoma* spp. was detected in *Se*. *khawi*, and *Ph*. *stantoni* [33, 34]. Recently, *Orbivirus* genus RNA was detected in *Idiophlebotomus* spp., *Ph*. *papatasi* (Scopoli) and *Se*. *khawi* [35]. In addition, the RNA of *Rhabdoviridae* family virus was detected in *Ph*. *papatasi* [36].

Most researchers have used adult morphology (cibarium, pigment patch, and spermathecae) and/or molecular methods to identify sand fly species in Thailand [26–37]. However, it is difficult to identify spermathecae in gravid females. Moreover, the systematic use of molecular tools/techniques to identify all sand flies collected from the field remains expensive. Therefore, morphological characteristics of sand fly eggs are of interest but there is currently no data on eggshell morphology for sand flies in Thailand. The objective of this study was to examine and characterize the ultrastructural morphology of eggs obtained from individual gravid female sand flies collected from Chiang Mai province, Thailand, using light microscopy (LM) and SEM.

## Materials and methods

### Sand fly collection and experimental design

Sand flies were collected on a weekly basis in August 2020 (four nights in total) in Doi Saket, Chiang Mai province, Thailand (E 18.892554, S 99.120376). Three CDC light-traps were set up in a cowshed from 6 PM to 6 AM. Collected sand flies were transferred to the laboratory of the Department of Parasitology, Faculty of Medicine, Chiang Mai University, for species identification and examination of eggs.

**Fig 1:**
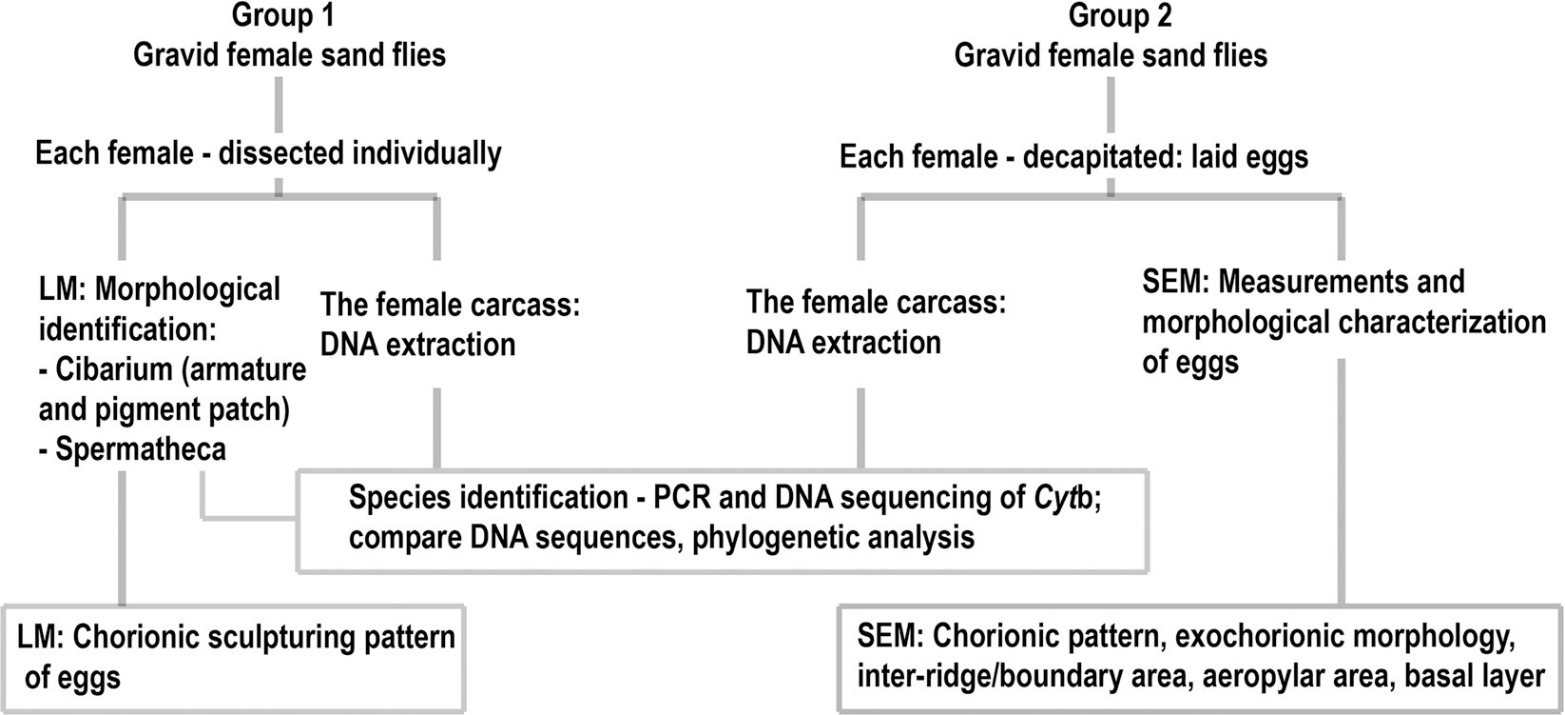
Experimental design for species identification of female sand flies and morphological examination and characterization of sand fly eggs.

The experimental design used for species identification of female sand flies and morphological examination and characterization of sand fly eggs is shown in Fig 1. Gravid females with similar morphology were separated into two groups. In Group 1, females were dissected individually for morphological identification using LM. Their carcasses were molecularly identified through DNA sequencing of the *cytochrome* b (*Cyt*b) gene. LM analysis of the chorionic sculpturing pattern of eggs in each gravid female was also performed. In Group 2, each gravid female was forced to lay eggs by decapitation on wet Whatman filter papers under a dissecting microscope. After laying eggs, DNA was extracted from the female carcass. DNA sequencing of the *Cyt*b gene for molecular identification of species was carried out through polymerase chain reaction (PCR). The eggs were left on the wet papers in a container for 24 h and subjected to SEM for measurements and morphological characterization including chorionic pattern, exochorionic morphology, inter-ridge/boundary area, aeropylar area, and basal layer.

### Light microscopy

The female samples in Group 1 were dissected in Phosphate Buffer Saline (PBS; 10 mM sodium phosphate, 145 mM sodium chloride, pH 7.2) under a dissecting binocular microscope. Only the female heads and abdominal segments 8–11 were detached and mounted in Hoyer’s medium. The morphological characteristics of the sand flies (cibarium, including armature and pigment patch; spermathecae) and eggs (chorionic sculpturing pattern) were examined and photographed using the LEICA DM1000 microscopy camera (Leica microsystems, Wetzlar, Germany). Species were identified using the adult morphological keys of Lewis [38, 39] as well as original descriptions and a recent taxonomical study by Depaquit and colleagues [28].

### Scanning electron microscopy and measurements of eggs

Eggs from individual females were fixed with 2.5% glutaraldehyde in 0.1 cacodylate buffer (pH 7.2) for 12 h at 4°C and washed twice for 10 min with PBS (pH 7.2). Next, samples were fixed with 1% osmium tetroxide in PBS for 1 h, and then dehydrated in a graded series of ethanol (50%, 70%, 90%, 95% for 10 min each and then twice with 100% ethanol for 30 min each), followed by critical point drying in liquid CO_2_ and coating with gold particles in a sputter-coating apparatus. The gold-coated preparations were examined under a scanning electron microscope, JEOL JSM-6610LV (JEOL, Tokyo, Japan), at 25–30 kV. SEM images of 30 eggs from at least three females in each species were used for egg measurements, including: length and width of egg, width and height of ridge, diameter and height of palisade unit, and diameter of tubercle. The values were presented as mean ± SD.

### DNA extraction, PCR, DNA sequencing and phylogenetic analysis

Each female carcass was transferred into 1.5 ml microcentrifuge tubes containing 200 μl of lysis buffer for gDNA extraction using a tissue DNA extraction kit (GeneJet Genomic DNA Purification kit, Thermo scientific, USA) following the manufacturer’s instructions. Genomic DNA was stored at 4°C until use. PCR of the *Cyt*b gene was performed using the N1N-PDR (5’—CAY-ATT-CAA-CCW-GAA-TGA-TA -3’) and C3B-PDR (5’—GGT-AYW-TTG-CCT-CGA-WTT-CGW-TAT-GA -3’) primers [40, 41]. The reaction was carried out in a final volume of 25 µl including 2.5 µl PCR buffer, 2.5 µl MgCl_2_, 2.5 µl dNTPs, 0.2 µl Taq polymerase, 0.4 µl forward, 0.4 µl reverse primers, 10.5 µl distilled water and 6 µl DNA template. PCR condition was performed with an initial denaturation step at 94°C for 3 min for 1 cycle, followed by 5 cycles: denaturation at 94°C for 30 sec, annealing at 40°C for 60 sec, extension at 68°C for 60 sec; and then 35 cycles of denaturation at 94°C for 60 sec, annealing at 45°C for 60 sec, extension at 68°C for 60 sec and a final extension step at 68°C for 10 min. PCR products were analyzed using electrophoresis on 1.5% agarose/1X TAE buffer gels, strained with ethidium bromide, and visualized under a UV transilluminator. PCRs were purified using the GeneJet PCR purification kit (Thermo Fisher scientific, USA) following the manufacturer’s instructions. The purified PCRs were sent to Macrogen Inc., Seoul, South Korea, for sequencing. A phylogenetic tree was built using the maximum likelihood method with the Tamura 3-parameter distance model in MEGA X [42].

## Results

### Species identification

**Fig 2:**
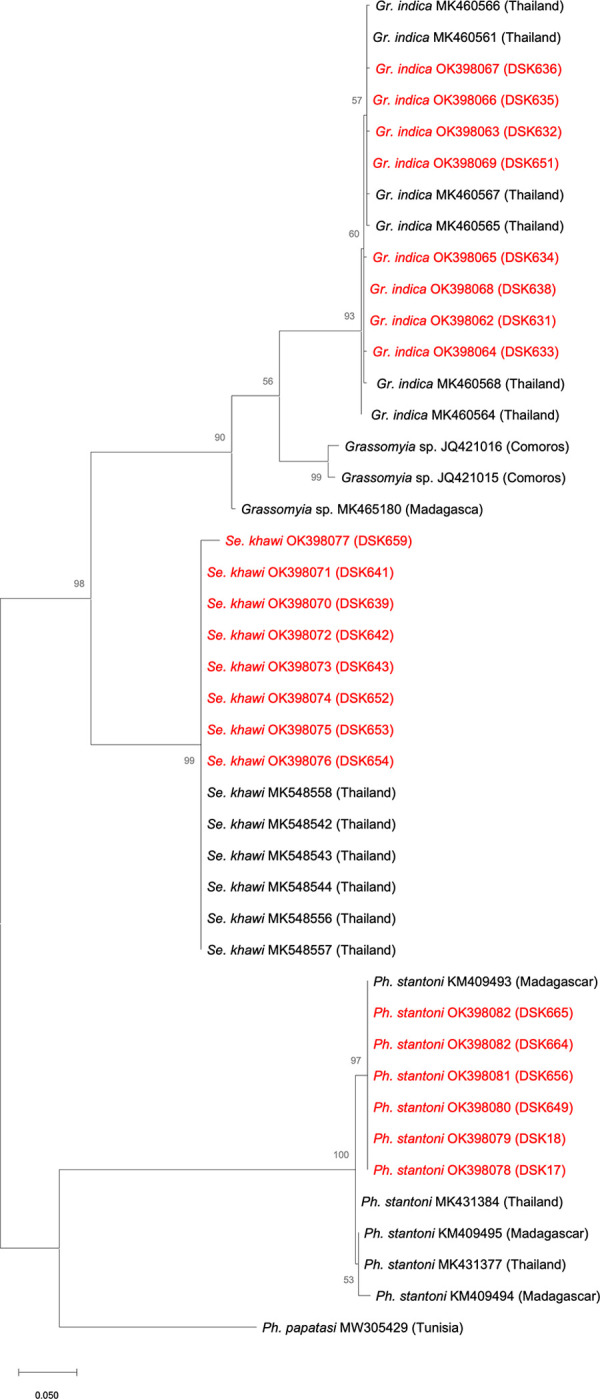
Maximum Likelihood tree based on the *Cyt*b sequences using the Tamura 3-parameter (T92) distance in MEGA X. The sequences obtained in the present study are written in red.

Twenty-two (22) gravid female sand flies were collected from the study area. They were examined and molecularly identified according to species. The *Cyt*b sequences of each sample were deposited in GenBank as listed in Table 1. Phylogenetic analysis revealed three species of sand flies, *i*.*e*., *Ph*. *stantoni*, *Se*. *khawi*, and *Gr*. *indica* (Fig 2).

**Table 1:** GenBank accession numbers of *Cyt*b sequences used to identify the species of sand flies in this study.

Species	Group 1 (No.)	GenBank accession number
*Ph*. *stantoni*	3	OK398078, OK398080, OK398081
*Se*. *khawi*	4	OK398070, OK398071, OK398072, OK398073
*Gr*. *indica*	3	OK398062, OK398063, OK398069
	Group 2 (No.)	
*Ph*. *stantoni*	3	OK398079, OK398082, OK398083
*Se*. *khawi*	4	OK398074, OK398075, OK398076, OK398077
*Gr*. *indica*	5	OK398064, OK398065, OK398066, OK398067, OK398068

### LM and SEM analyses

The eggshell structures of the three sand fly species, *Ph*. *stantoni*, *Se*. *khawi*, and *Gr*. *indica*, were examined and described. In general, all eggs were elongated, elipsoidal in shape with one side slightly flattened and rounded polar regions. In this study, only an aeropyle of *Ph*. *stantoni* was observed at the anterior pole of eggs.

Two chorionic patterns of eggs were found: (1) an unconnected parallel ridges pattern combined with a reticular pattern in *Ph*. *stantoni* and (2) a polygonal pattern in *Se*. *khawi* and *Gr*. *indica*. The details of the eggshell structures of the three sand fly species are described below.

#### Phlebotomus stantoni

**Fig 3:**
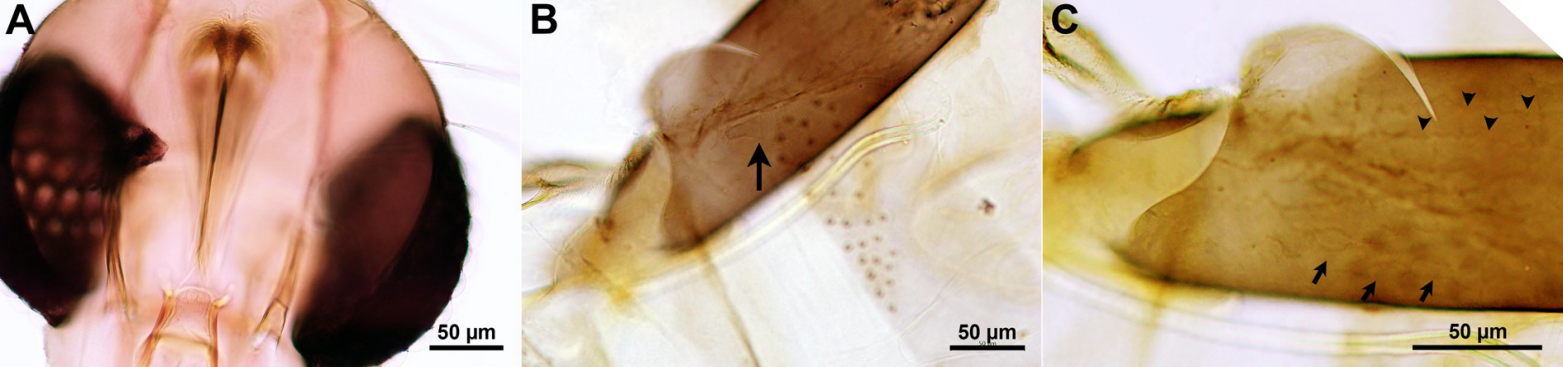
*Phlebotomus stantoni*. A. LM of head showing ciberial teeth; B. LM showing a spermatheca (arrow); C. LM showing a chorionic pattern of the egg with unconnected parallel ridges (arrowheads) and reticular pattern (arrows).

*Phlebotomus stantoni* has a cibarium with approximately 15 pointed denticles of various lengths, which are irregularly arranged. The two or three median denticles are longer and stouter than the others and without a pigment patch (Fig 3A). The species has a fusiform spermatheca with 15 or 16 rings. The neck is thick and short, the head more or less oblong. The duct is striated and the common duct is very long, about 1.5 times the length of the individual ones, with a thick wall sclerotized at its basal part (Fig 3B). LM analysis of eggs in *Ph*. *stantoni* gravid females showed two patterns: unconnected parallel ridges and a reticular pattern on the same egg (Fig 3C).

*Egg size*. length = 301.20 ± 2.11 μm; width = 83.65 ± 1.63 μm; N = 30.

**Fig 4:**
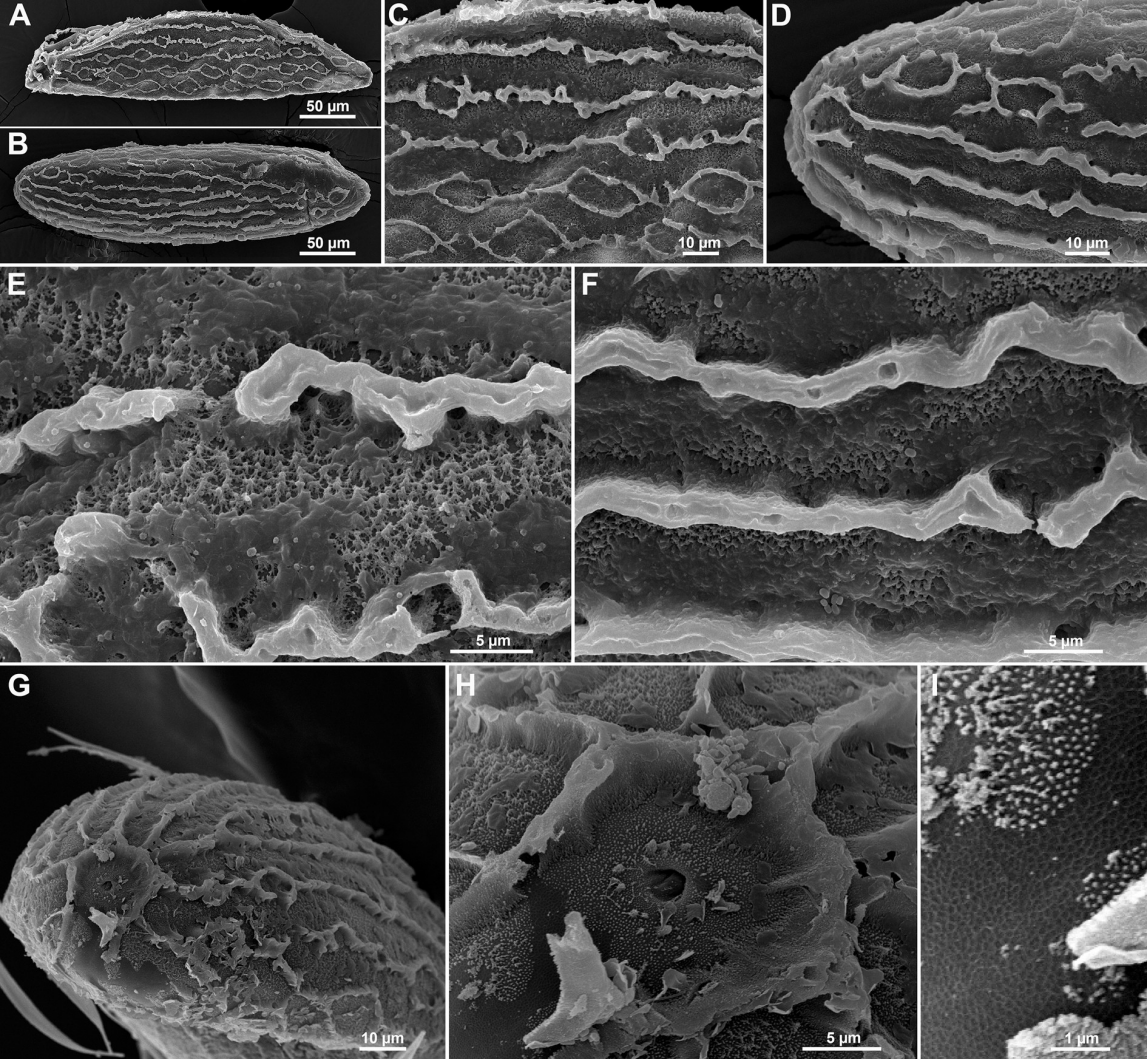
Ultrastructural morphology of *Ph*. *stantoni* eggs. A, B. SEM of whole eggs showing chorionic sculpturing with unconnected parallel ridges and a reticular pattern on the same egg; C, D. Details of the chorionic pattern; E, F. Coarsely arranged fibrous material which formed minute protuberances between ridges; G. Anterior pole showing the aeropylar area with one aeropyle opening; H. Higher magnification of the aeropylar area delimited by a single non-columnar circular ridge; I. A reticular basal layer near the aeropylar area.

*Chorionic pattern*. The chorionic pattern of *Ph*. *stantoni* combines two patterns on the same egg, unconnected parallel ridges and a reticular pattern (Fig 4A–4D).

*Exochorionic morphology*. Ridges are formed by double non-columnar palisades that project from the surface of the eggshell. In some areas, the ridges are fused, forming an unconnected parallel ridges pattern. In other areas, the ridges exist separately thereby forming a reticular pattern. Each ridge is 3.37 ± 0.79 μm in width and 5.88 ± 0.35 μm in height (Fig 4C–4F).

*Inter-ridge/boundary area*. Fibrous material in the inter-ridge area is coarsely arranged with minute protuberances covering the basal layer (Fig 4E and 4F).

*Aeropylar area*. One aeropyle opening is present. The aeropyle is surrounded by randomly distributed protuberances and covered by very small dots (Fig 4G and 4H). The aeropylar area is delimited by a single non-columnar circular ridge.

*Basal layer*. A wide reticular mesh conformation of the basal layer near the aeropyle opening is present (Fig 4I).

#### Sergentomyia khawi

**Fig 5:**
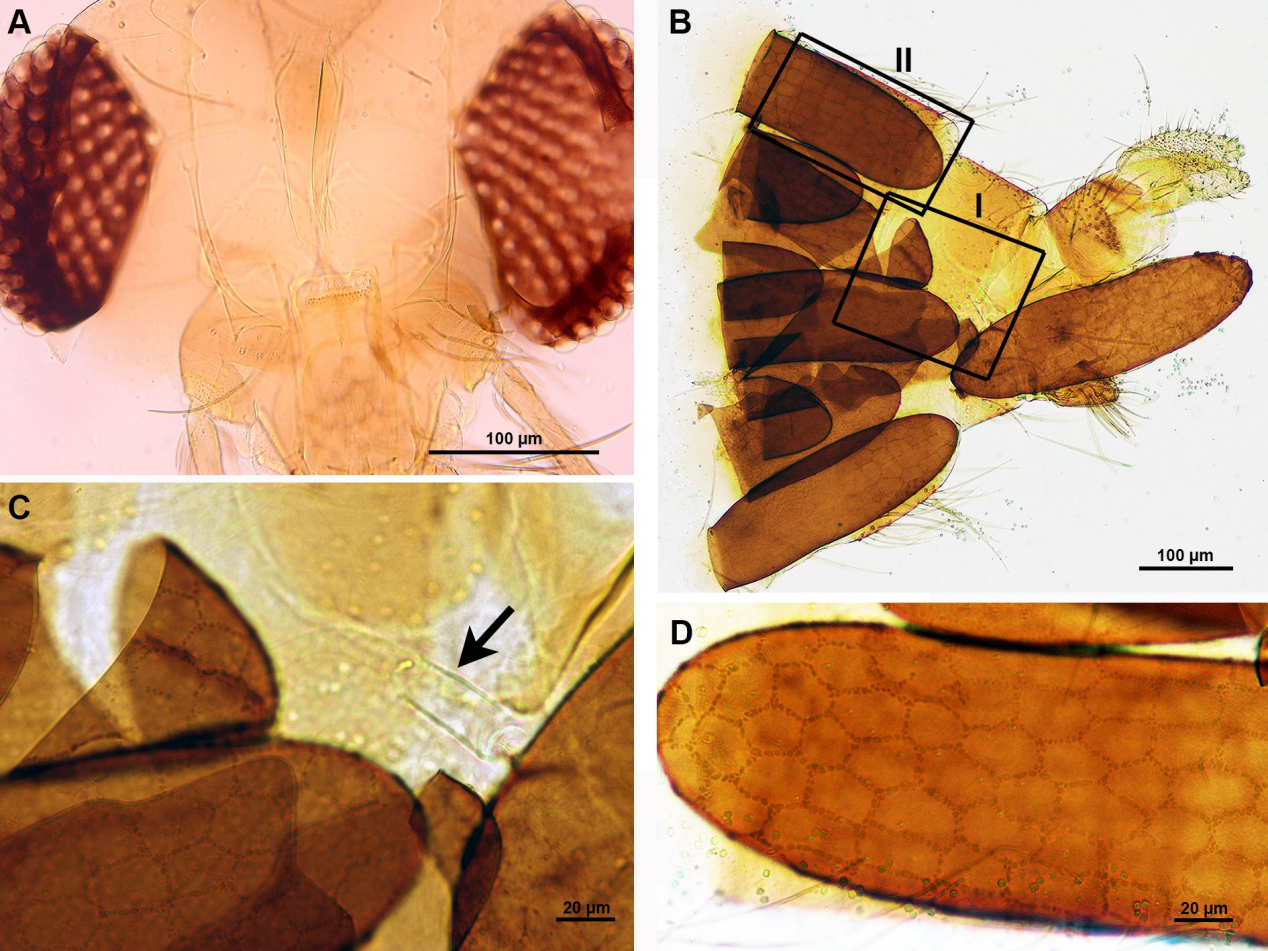
*Sergentomyia khawi*. A. LM of a head showing the cibarial teeth; B. Posterior abdominal region of a gravid female sand fly with eggs inside; C. Higher magnification of the area of box I in B showing a spermatheca (arrow); D. Higher magnification of the area of box II in B showing the polygonal pattern of the sand fly egg.

*Sergentomyia khawi* has a cibarium containing 10–12 hind teeth with two or three rows of fore teeth with a light pigment patch (Fig 5A). It has a narrow shaped spermatheca with a few wrinkles, with a knob in a deep narrow pit (Fig 5C). LM analysis of eggs in *Se*. *khawi* gravid females reveals polygonal (rectangular, pentagonal, or hexagonal) patterns of the chorion (Fig 5B–5D).

*Egg size*. length = 289.88 ± 1.78 μm; width = 68.85 ± 1.20 μm; N = 30.

**Fig 6:**
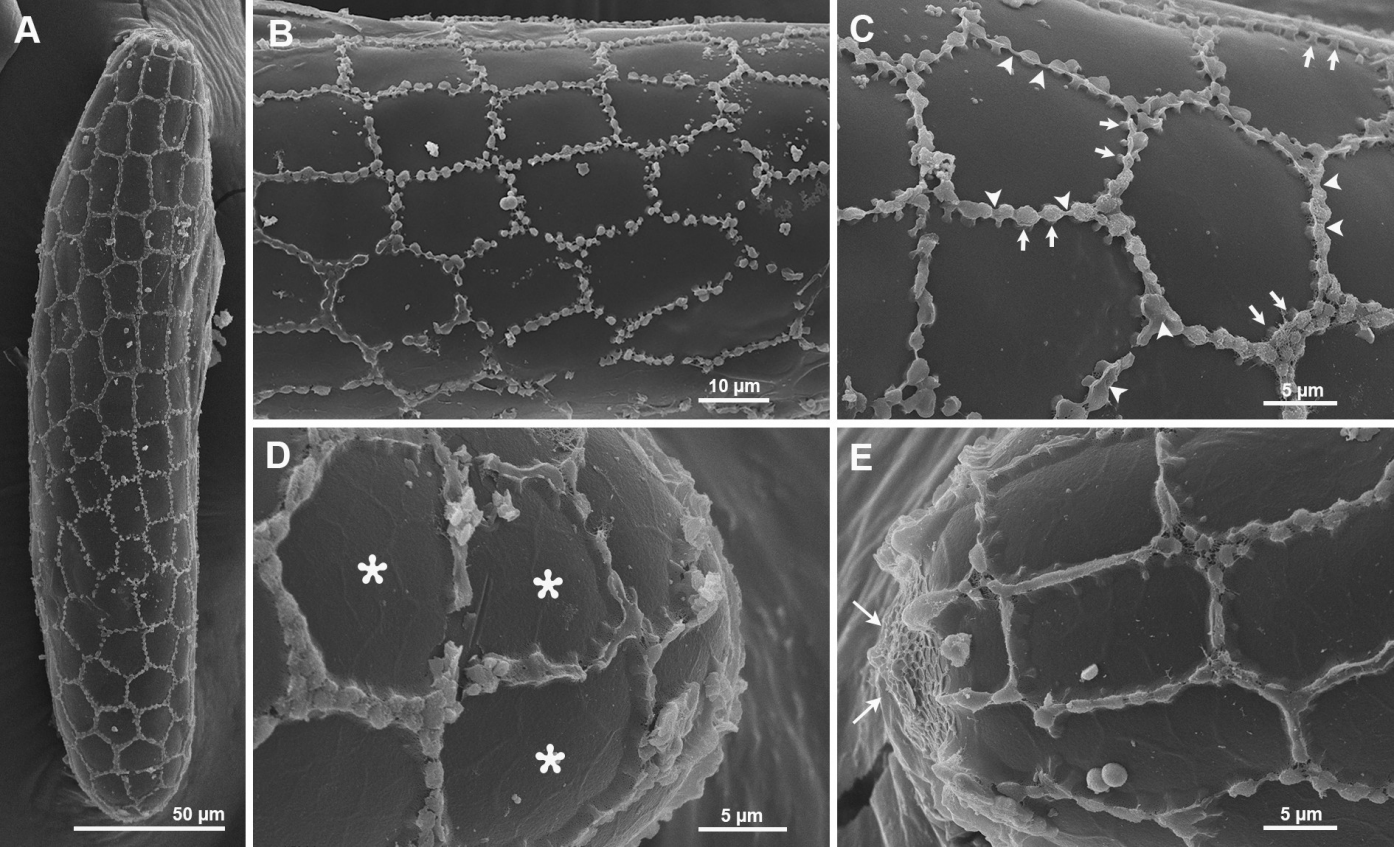
Ultrastructural morphology of *Se*. *khawi* eggs. A. SEM of a whole egg showing chorionic sculpturing with a polygonal pattern; B. Details of the general appearance of the chorionic sculpturing; C. High magnification image showing ridges formed by the intersection of single or double parallel series of rounded palisade units (arrows). Palisade units are linked or united at the top (arrowheads); D. Details of finely arranged fibrous material as a compact coat in inter-ridge areas (asterisks); E. Chorionic pattern near the pole and a thick reticular mesh conformation (arrows).

*Chorionic pattern*. The ornamentation of the chorionic sculpturing pattern is polygonal (rectangular, pentagonal, and hexagonal). The polygonal pattern is due to ridges that are continuous, forming alternating transversal rows of polygonal cells (Fig 6A–6E).

*Exochorionic morphology*. Ridges are formed by the intersection of a single or double parallel series of rounded palisade units that project from the surface of eggshell. The diameter and height of each palisade unit are 1.26 ± 0.21 μm and 0.8 ± 0.14 μm, respectively. Almost all palisade units are linked or united at the top (Fig 6B and 6C).

*Inter-ridge/boundary area*. Finely arranged fibrous material as a compact coat covering the basal layer of the egg is present in the inter-ridge area (Fig 6D).

*Aeropylar area*. A wide reticular mesh conformation is present at the pole (Fig 6E).

#### Grassomyia indica

**Fig 7:**
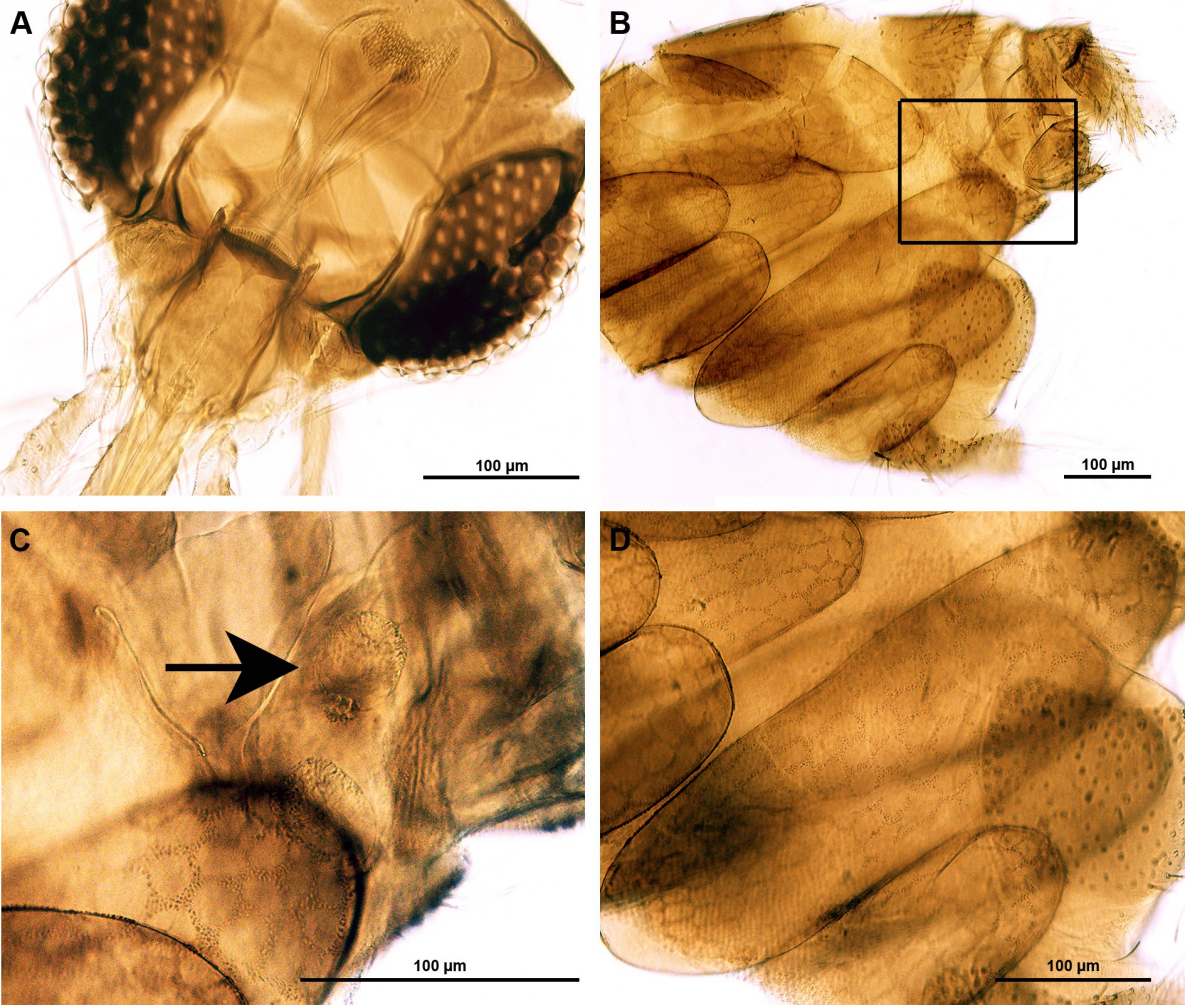
*Grassomyia indica*. A. LM of head showing cibarial teeth; B. Posterior abdominal region of a gravid female sand fly with eggs inside; C. Higher magnification from the area of box in B showing spermathecae (arrows); D. Polygonal pattern of the sand fly eggs.

*Grassomyia indica* has a cibarium with 29–30 teeth along a convex curved line and a dark brown pigment patch with a straight anterior margin (Fig 7A). It has a round finely speculate capsule spermatheca with minute projections at its tip (Fig 7C). LM analysis of eggs inside *Gr*. *indica* females reveals polygonal patterns (rectangular, pentagonal, or hexagonal) of the chorionic sculpturing (Fig 7B–7D).

*Egg size*. length = 265.63 ± 0.99 μm; width = 55.61 ± 0.91 μm; N = 30.

**Fig 8:**
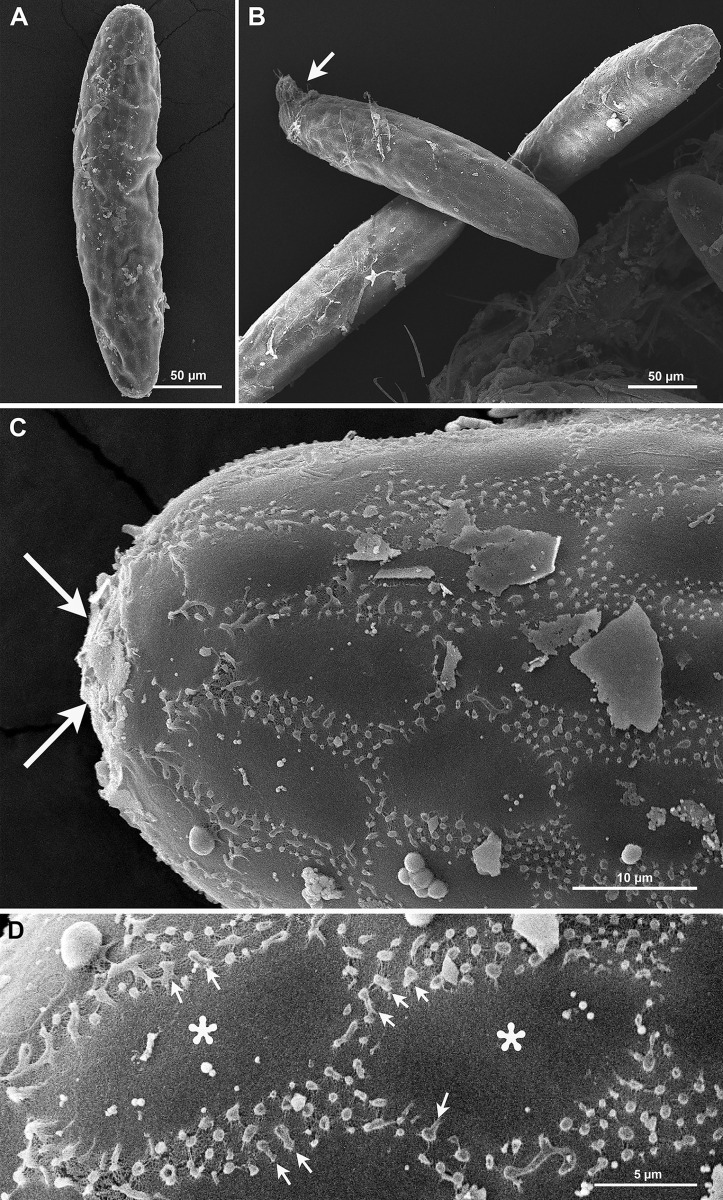
Ultrastructural morphology of *Gr*. *indica* eggs. A. SEM of whole eggs showing chorionic sculpturing with a polygonal pattern; B. Larval head protruding from the anterior pole (arrow); C. Details of the general appearance of the chorionic pattern and a thick coat material at the pole (arrows); D. High magnification image showing boundaries of polygonal shapes formed by intersection of 2–5 lines of small randomly arranged tubercles. Some tubercles project from the surface of the eggshell (arrows). Details of finely arranged fibrous material as a compact coat in inter-boundary areas (asterisks).

*Chorionic pattern*. The ornamentation of the chorionic pattern is polygonal, mostly rectangular and pentagonal shapes, occasionally hexagonal shapes (Fig 8A–8D).

*Exochorionic morphology*. Boundaries of the polygonal shapes are formed by the intersection of 2–5 lines of small randomly arranged tubercles. Some tubercles project from the surface of the eggshell. The average of diameter of the tubercles is 0.68 ± 0.14 μm. No tubercles are linked or united at the top (Fig 8C and 8D).

*Inter-ridge/boundary area*. Fibrous material in the inter-ridge area is finely arranged as a compact coat covering the basal layer (Fig 8D).

*Aeropylar area*. A thick coat material is present at the pole (Fig 8C).

Table 2 summarizes the important details of ultrastructural morphology of Old World sand fly eggs examined by SEM.

**Table 2:** Important details of Old World sand fly eggs examined by SEM.

Species	Chorionic pattern	Exochorionic morphology	Inter-ridge/boundary area	Number of aeropyle, Aeropylar area	Basal layer	Reference(s)
Genus *Phlebotomus*						
Subgenus *Anaphlebotomus*						
*Ph*. *stantoni*	Unconnected parallel ridges and reticular	Ridges formed by double non-columnar palisades, Width = 2.1–4.2 μm and Height = 5.5–6.3 μm	Coarse minute protuberances	1, aeropyle surrounded by randomly distributed protuberances and covered by very small dots, single non-columnar circular ridge	Reticular	This study
Subgenus *Euphlebotomus*						
*Ph*. *argentipes* Annandale & Brunetti in Annandale	Connected parallel ridges (hexagonal in some areas)	Ridges formed by single column palisade series	Rough, having intercalated networks	ND^a^	ND	[25]
Subgenus *Phlebotomus*						
*Ph*. *papatasi* Scopoli	Connected parallel ridges (reticular in some areas)	Ridges formed by double column palisade series, Height = 4–4.5 μm	Coarse minute protuberances, rough, having uniform small granular protuberances	2, double non-columnar circular ridge, sub-divided by a transverse ridge into two semi-circular areas	Reticular	[22, 23, 25]
*Ph*. *duboscqi* Neveu-Lemaire	Reticular	Ridges formed by double column palisade series, Height = 3.5–4 μm	Coarse minute protuberances	2, single non-columnar circular ridge, sub-divided by transverse ridge into two semi-circular areas	Reticular	[23]
Subgenus *Larroussius*						
*Ph*. *perniciosus* Newstead	Unconnected parallel ridges	Ridges formed by double column palisade series, Height = 2–2.5 μm	Fine, as a compact coat	2, single non-columnar circular ridge, sub-divided by a transverse ridge into two semi-circular areas	Rough	[23]
*Ph*. *perfiliewi* Parrot	Unconnected parallel ridges	Ridges formed by single thin column palisade series, Height = 1.5–2 μm	Fine, as a compact coat	2, single non-columnar circular ridge, sub-divided by a transverse ridge into two semi-circular areas	Rough	[23]
*Ph*. *pedifer* Lewis, Mutinga & Ashford	Parallel ridges	ND	ND	ND	ND	[24]
*Ph*. *aculeatus* Lewis, McMillan & Ashford	Variations: polygonal, beehive cell, parallel ridges	ND	ND	ND	ND	[24]
*Ph*. *major* Annandale	Polygonal	Ridges formed by columns of granular cells arranged to form a palisade	ND	ND	ND	[25]
Subgenus *Synphlebotomus*						
*Ph*. *celiae* Minter	Reticular	ND	ND	ND	ND	[21]
*Ph*. *martini* Parrot	*Kenya*: Connected parallel ridges *Ethiopia*: Reticular	ND	ND	ND	ND	[21]
	Variations: parallel ridges, hexagonal, mountainlike, irregular pattern	ND	ND	ND	ND	[20]
Genus *Sergentomyia*						
Subgenus *Rondanomyia*						
*Se*. *khawi*	Polygonal	Ridges formed by single or double parallel series of rounded palisade units, Diameter = 0.7–1.5 μm and Height = 0.6–1.0 μm	Fine, as a compact coat	ND, a wide reticular mesh conformation	ND	This study
*Se*. *zeylanica* (Annandale)	Polygonal	Solid granules of various sizes, arranged in a column to form a palisade, ridges appear to be wide and solid, uniform cord like structures	ND	ND	ND	[25]
*Se*. *kirki* (Parrot)	Volcano-like	ND	ND	ND	ND	[20]
Subgenus *Sergentomyia*						
*Se*. *garnhami* (Heisch, Guggisberg & Teesdale)	Parallel ridges	ND	ND	ND	ND	[20]
*Se*. *bedfordi* (Newstead)	Polygonal	ND	ND	ND	ND	[20]
Genus *Grassomyia*						
*Gr*. *indica*	Polygonal	Boundaries formed by 2–5 lines of small randomly arranged tubercles, Diameter = 0.3–0.8 μm	Fine, as a compact coat	ND, covered by a thick coat material	ND	This study

^a^ND = not described

## Discussion

Eggs of approximately ten species of the genus *Phlebotomus* belonging to four subgenera, i.e., *Euphlebotomus*, *Larroussius*, *Phlebotomus*, and *Synphlebotomus* [20–25] and five species in the genus *Sergentomyia* belonging to two subgenera, i.e., *Rondanomyia* and *Sergentomyia* [20, 25] have been previously examined by SEM. In these past studies, the sand fly eggs were obtained from females reared in laboratories (laboratory strains) [22, 23, 25] or collected in the field. The females were allowed to feed on animal blood in the laboratory to favor egg development [20, 24, 25] but no molecular methods for species identification were mentioned. In the case of field-caught female sand flies, sibling species which are morphologically indistinguishable but reproductively isolated may be obtained together. Rogo and colleagues [24] observed that eggs laid by an individual female of *Ph*. *martini* were identical but those laid by different females of the same species (identified by morphology) were not. In addition, eggs of *Ph*. *martini* females collected from different origins, such as Kenya and Ethiopia, showed different chorionic patterns [20, 21].

Therefore, in this study, both morphological identification keys of female sand flies and DNA sequences of the *Cyt*b gene were used for species identification and confirmation. In addition, a systematic collection of egg batches from individual females was performed by decapitating the gravid females. It is the first time this method was used in sand flies from Thailand and it allowed us to obtain synchronized eggs for SEM analysis. The species of the females and eggs were reliable and consistent by using these different approaches.

Our results show that the egg morphology of the three confirmed species, *Ph*. *stantoni*, *Se*. *khawi* and *Gr*. *indica*, followed the shape of eggs of all the previously described species: they have a long and oval shape with one side slightly flattened and rounded polar regions. Five details in the ultrastructural morphology of sand fly eggs including chorionic pattern, exochorionic morphology, inter-ridge/boundary area, aeropylar area and basal layer were examined and characterized by SEM. However, only complete details of *Ph*. *stantoni* eggs were obtained. The chorionic sculpturing of *Ph*. *stantoni* (subgenus *Anaphlebotomus*) eggs differs from that of sand flies in other subgenera. We demonstrated for the first time that the chorionic pattern of *Ph*. *stantoni* eggs combines “unconnected parallel ridges” and a “reticular” pattern on the same egg. In *Ph*. *argentipes* (subgenus *Euphlebotomus*) and *Ph*. *papatasi* (subgenus *Phlebotomus*), although the chorionic pattern of eggs has been grouped under “connected parallel ridges”, a reticular pattern was observed in some areas on the eggs [25]. A combination of double parallel connected ridges and reticular pattern has also been reported in *Lu*. *verrucarum* [8]. Moreover, a variation of the chorionic sculpturing pattern among sand flies in the genus *Phlebotomus* has been reported. For example, in the subgenus *Larroussius*, the pattern of *Ph*. *perniciosus* and *Ph*. *perfiliewi* eggs is “unconnected parallel ridges” [23] whereas various patterns (polygonal, beehive cell, and parallel ridges) have been reported in *Ph*. *aculeatus* [24]. Furthermore, in the subgenus *Synphlebotomus*, the chorionic pattern of *Ph*. *celiae* eggs is “reticular” while *Ph*. *martini* sand flies from different countries (Kenya and Ethiopia) have different chorionic patterns of eggs, such as “connected parallel ridges”, “reticular”, “mountain-like”, irregular patterns [20, 21, 24].

In the genus *Sergentomyia*, a polygonal pattern is quite common, however, a “volcano-like” pattern and “parallel ridges” have been reported in *Se*. *kirki* (subgenus *Rondanomyia*) and *Se*. *garnhami* (subgenus *Sergentomyia*), respectively [20]. The chorionic pattern of *Se*. *khawi* and *Gr*. *indica* from Thailand is polygonal but their exochorionic morphology and aeropylar area are different. The exochorionic morphology of *Gr*. *indica* is unique. Boundaries of the polygonal pattern formed by 2–5 lines of small randomly arranged tubercles in *Gr*. *indica* were noted and described for the first time. The aeropylar area of *Se*. *khawi* is covered by a wide reticular mesh conformation whereas the aeropylar area of *Gr*. *indica* is covered by a thick coat material. Differences in exochorionic morphology, the inter-ridge/boundary area, the aeropylar area (including the number of aeropyles) and basal layer of some sand fly species in the genus *Phlebotomus* in the New World have been reported [10, 12, 15–19, 22, 23, 25]. Therefore, the chorionic pattern together with other important details of the ultrastructural morphology may be useful for developing morphological identification keys of eggs as an additional tool to distinguish species of sand flies. More information on egg ultrastructural morphology of sand flies in the Old World and the New World with correct molecular genetic information are required for further analysis to ascertain their taxonomic values before any conclusion can be drawn.

Although the use of SEM has significantly improved the characterization of the ultrastructural morphology of sand fly eggs, in this study, the chorionic sculpturing of the eggs of *Ph*. *stantoni*, *Se*. *khawi*, and *Gr*. *indica*, were observed by LM. We demonstrate that in the case of gravid females (whose spermathecae is obscured by eggs), the chorionic sculpturing of eggs is useful to identify these three species. In this study, the developmental stages of eggs in the gravid female sand flies could not be estimated since these samples were taken from females caught in the wild. Our results showed that in LM, the chorionic pattern of eggs inside the females of each species was the same as shown in SEM. The egg samples for SEM were allowed to fully melanise for 24 h before SEM processing. This suggests that the chorionic patterns of fully mature eggs were present in the abdomens of gravid females of these three sand fly species. In *Lu*. *longipalpis*, fully melanised eggs have been observed in the abdomens of gravid sand flies. It highlights that sand fly eggs can fully melanise prior to oviposition [43]. Therefore, it is interesting to further investigate chorionic patterns in each developmental stages of sand fly eggs of each species. In addition, further studies on eggshell morphology of sand flies from other areas and other species are needed to compare eggshell patterns as the environments of oviposition sites influence the exochorion of sand fly eggs [4]. At least two species in the same genus in the Old World, i.e., *Phlebotomus*, *Sergentomyia*, *Grassomyia*, *Idiophlebotomus*, *Chinius*, *Parvidens*, *Spelaomyia* and *Spelaeophlebotomus* should be investigated to evaluate their informativity for classification of sand flies.

In conclusion, the ultrastructural morphology of the eggshells of *Ph*. *stantoni*, *Se*. *khawi*, and *Gr*. *indica* sand flies was described for the first time. Our findings indicate that the chorionic sculpturing pattern together with other important details of the ultrastructural morphology can be used to develop morphological identification keys of eggs. These can serve as an additional tool to distinguish species of sand flies. The systematic collection of egg batches from individually gravid females by decapitating allowed us to obtain synchronized eggs for SEM analysis. In addition, insect species were confirmed using both *Cyt*b sequencing as well as adult morphological identification keys. Together, these methods provided useful information for sand fly ootaxonomy. Furthermore, the chorionic sculpturing of the eggs of these three species observed by LM is useful for species identification in the case of gravid females with spermathecae obscured by eggs.
